# Synergy of alanine and gentamicin to reduce nitric oxide for elevating killing efficacy to antibiotic-resistant *Vibrio alginolyticus*

**DOI:** 10.1080/21505594.2021.1947447

**Published:** 2021-07-12

**Authors:** Su-fang Kuang, Yue-tao Chen, Jia-jie Chen, Xuan-xian Peng, Zhuang-gui Chen, Hui Li

**Affiliations:** a State Key Laboratory of Bio-Control and the Third Affiliated Hospital, School of Life Sciences, Southern Marine Science and Engineering Guangdong Laboratory Zhuhai, Sun Yat-sen University, University City, Guangzhou, China; b Laboratory for Marine Fisheries Science and Food Production Processes, Qingdao National Laboratory for Marine Science and Technology, Qingdao, China

**Keywords:** *V. alginolyticus*, Ala, Gent, nitric oxide, NOS, metabolomics

## Abstract

The present study explored the cooperative effect of both alanine (Ala) and gentamicin (Gent) on metabolic mechanisms by which exogenous Ala potentiates Gent to kill antibiotic-resistant *Vibrio alginolyticus*. To test this, GC-MS-based metabolomics was used to characterize Ala-, Gent- and both-induced metabolic profiles, identifying nitric oxide (NO) production pathway as the most key clue to understand metabolic mechanisms. Gent, Ala and both led to low, lower and lowest activity of total nitric oxide synthase (tNOS) and level of NO, respectively. NOS promoter L-arginine and inhibitor N^G^-Monomethyl-L-arginine inhibited and promoted the killing, respectively, with the elevation and decrease of NOS activity and NO level. The present study further showed that CysJ is the enzyme-producing NO in *V. alginolyticus*. These results indicate that the cooperative effect of Ala and Gent causes the lowest NO, which plays a key role in Ala-potentiated Gent-mediated killing.

## Introduction

The rise of bacterial antibiotic resistance and insufficient development of new antibiotics to antibiotic-resistant pathogens have created a global health crisis. The crisis is putting the world at the risk of a post antibiotic era, where the existing antibiotic-mediated killing efficacy is lost [1–3]. Thus, development of new approaches that readjust the killing efficacy to control these antibiotic-resistant pathogens demands immediate attention.

Metabolome-reprogramming is a recently developed approach to restore the killing efficacy of the existing antibiotics [4–6]. A line of evidence has showed that antibiotic-resistant and antibiotic-sensitive bacteria have antibiotic-resistant and antibiotic-sensitive metabolomes, respectively. Crucial biomarkers such as glucose, alanine (Ala), and glycine identified from differential metabolomes between the antibiotic-resistant and antibiotic-sensitive metabolomes reprogram the antibiotic-resistant metabolome to the antibiotic-sensitive metabolomes [7–10]. The reprogramming reverts bacterial defensive capability against the existing antibiotics, promotes the drugs uptake and thereby potentiates the existing antibiotic-mediated killing efficacy since limitation of antibiotic uptake is a widely accepted mechanism by which bacteria resist existing antibiotics [5]. The approach has been demonstrated in Ala-, glucose-, glutamate-enabled killing of antibiotic-resistant *Edwardsiella tarda, Escherichia coli* and *Vibrio alginolyticus* by the existing aminoglycoside antibiotics kanamycin and gentamicin (Gent) in addition to ampicillin, ceftazidime and balofloxacin [5–9]. Further analysis shows that modulation of the central carbon metabolism and energy metabolism by the crucial biomarkers plays a role in the reprogrammed metabolomes [5,6,9–11]. However, whether antibiotics have a synergetic effect with the crucial biomarkers in the reprogramming is unknown.

*V. alginolyticus* is a zoonotic pathogen that affects human health and fish breeding, causing fatal disease and severe economic loss, respectively. As antibiotics are widely used to treat infectious disease caused by the bacterium, antibiotic-resistant *V. alginolyticus* isolates have frequently been reported. Among them, 38–82.1% isolated strains are resistant to Gent [12–14]. Very recently, we have used GC-MS-based metabolomics to explore *V. alginolyticus* Gent-resistant metabolome, where glucose and the reduced redox state were identified as the most downregulated metabolite and the metabolic phenotype, respectively [8]. We have further showed that exogenous glucose reprograms the metabolic state and thereby promotes Gent uptake and elevates Gent-mediated killing [8]. However, we investigated the metabolome reprogrammed by the crucial biomarker glucose, but did not explore that reprogrammed by the synergistic effect of both glucose and Gent. Antibiotics also affect bacterial metabolism [15,16]. Therefore, it is interesting to understand whether the synergy plays a role in the metabolites-potentiated Gent-mediated killing.

In our reported *V. alginolyticus* Gent-resistant metabolome, besides glucose, Ala is also a depressed metabolite belonging to the most impacted metabolic pathway alanine, aspartate, and glutamate metabolism [8]. Thus, the present study used GC-MS-based metabolomics to characterize metabolomes induced by Ala, Gent and both. Our results showed that the synergy of Ala and Gent reduces arginine (Arg) biosynthesis, causes decrease of nitric oxide (NO) and thereby promotes Gent-mediated killing. In our knowledge, this is the first report that the reprogrammed metabolome caused by the synergy of both metabolite and antibiotic changes NO metabolism to potentiate the existing antibiotic-mediated killing.

## Methods

### Bacterial strains and culture conditions

The bacterial strains used in the current study were *V. alginolyticus* ATCC33787, Gent-resistant *V. alginolyticus* V2G01 (VA-R_GEN_) and clinically isolated *Vibrio* spp. strains, which were obtained from the collection of our laboratory. These bacteria were grown at 30°C in 50 mL LB broth (1% bacterial peptone, 0.5% yeast extract and 3% NaCl) overnight and harvested by centrifugation at 6,000 × g for 3 min.

### Antibiotic bactericidal assay

Antibiotic bactericidal assay was carried out as described previously [17]. The well-cultured bacterial cells described above were collected and washed three times and then suspended in M9 with 10 mM acetate, 2 mM MgSO_4_ and 100 mM CaCl_2_ to 0.6 of OD_600_ nm. Ala or/and Gent or other aminoglycoside antibiotics were added and incubated at 30°C, 200 rpm for 6 h. Arg, N^G^-Monomethyl-L-arginine (L-NMMA, Monoacetate Salt, Beyotime S0011) and H_2_O_2_ were added if desired. To determine colony-forming units (CFU) per mL, 100 μL of samples were 10-fold serially diluted and an aliquot of 10 μL of each dilution was spotted on the LB agar plates and cultured at 30°C for 8 h. Notably, in the detection of H_2_O_2_-mediated killing, 2,500 units/mL catalase (Sigma, C1345) was added to quench H_2_O_2_ before the dilution for plating. Only those dilutions yielding 20–200 colonies were enumerated to calculate colony-forming units (CFU). The percent survival is determined by dividing the CFU obtained from the treated sample by the CFU obtained from the control.

### Metabolic profiling

Metabolic profiles were detected by GC-MS-based metabolomics as described previously [9]. Samples from different treatments with Ala or/and Gent were prepared as follows: Bacteria were collected, washed with saline, and adjusted to an OD_600_ nm value of 1.0 to remove M9 medium. Then, 10-mL resuspension was collected by centrifugation at 2,300 × g at 4°C and transferred into 1.5 mL QSP microtube, immediately quenched with −80°C pre-cooled methanol (Sigma). Bacterial metabolites were extracted after repeated freeze-thaw cycles of liquid nitrogen. The 0.1 mg/mL ribose was added as the internal standard and the resuspension was sonicated for 10 min at the 200 W working power. The supernatant was separated by centrifugation at 4°C and 14,200 × g for 10 min and placed in a 37°C vacuum centrifuge dryer (Labconco, USA) to evaporate the methanol. Dry extracts were performed on GC-MS analysis. The dried samples were added to 80 μL of 20 mg/mL methoximation-pyridine hydrochloride (Sigma-Aldrich). After sonication, the reaction was performed at 37°C for 3 h. Subsequently, 80 μL of N-methyl-N-trimethylsilyltrifluoroacetamide (MSTFA, Sigma) was added and the reaction was performed at 37°C for 30 min. GC-MS data were detected by Thermo Scientific Trace DSQ II.

### GC-MS data analysis

Statistical analysis was performed as described previously [9]. In brief, mass fragmentation spectrum was analyzed by XCalibur software (Thermo fisher, version 2.1) to identify compounds by matching data with the National Institute of Standards and Technology (NIST) library and NIST MS search 2.0 program. The data were normalized according to total amount correction and standardized data containing metabolites, retention times, and peak areas, and used for further metabolomics analysis. The software IBM SPSS Statistics 19 was used to analyze the significant difference of the standardized data and the metabolites with differences were selected (P-value < 0.05). The R software (R × 64 3.6.1) was used for cluster analysis. Principal component analysis and S-plot analysis were performed by SIMCA-P + 12.0 software (version 12; Umetrics, Umea, Sweden) and the metabolic pathway was done with MetaboAnalyst 4.0 enrichment. Interactive Pathways (iPath) analysis was carried out by iPath3.0 (https://pathways.embl.de/).

### Cloning, expression, and purification of NO synthase homologous protein/gene

Homologous nitric oxide synthase (NOS; CysJ, AT730_09320) in *V. alginolyticus* ATCC33787 was searched against GenBank. Briefly, the NOS protein sequences from human (NP_000616), mouse (NP_035057), *Pyricularia oryzae* 70–15 (EHA54937), *Ophiocordyceps sinensis* CO18 (EQL02386), *Pseudomonas putida* GB-1 (ABY96813), and *Rheinheimera* sp. D18 (QBL10471) were compared with all protein sequences of *V. alginolyticus*. The highest matching protein was selected as a NOS-like protein candidate, which led to selection of CysJ (AT730_09320). For cloning of *cysJ*, primers were designed for PCR amplification (Table S1). *cysJ* gene was cloned into pET-32a expression vector with His-tag and recombinant plasmid was transformed into *E. coli* BL21 (DE3). For CysJ protein expression and purification, a colony of *E. coli* BL21 (DE3) containing the recombinant plasmid was cultured in 5 mL of LB medium with of 100 μg/mL ampicillin at 37°C with shaking for 12 h. The cultures were diluted in 1: 100 to 500 mL LB medium supplemented with ampicillin and incubated at 37°C until the OD_600_ nm reached at 0.5. Then, 0.1 mM isopropyl-D-thiogalactopyranoside was added to induce recombinant protein at 20°C for 12 h. Subsequently, bacteria were harvested by centrifugation at 6,000 × g and resuspended in a 50 mM Tris-HCl (pH 7.4). The resulting cells were disrupted by high pressure (23 kpsi) using a cell disruptor (Constant Systems Ltd. Daventry, UK) and supernatant was obtained by centrifugation at 6,000 × g for 30 min at 4°C. The supernatant was applied onto a nickel-affinity column and incubated at 4°C for 2 h. After protein binding, the column was washed with 10 volumes of washing buffer (1× PBS buffer with 5 mM imidazole) to remove unbound protein. Recombinant protein (rCysJ) was eluted with five volumes of elution buffer (1× PBS buffer with 50 mM imidazole). Finally, purity of recombinant protein was analyzed by SDS-PAGE electrophoresis and concentration was quantified by BCA protein concentration determination kit (Beyotime, P0009).

### Enzyme activity determination

Determination of pyruvate dehydrogenase (PDH), alpha-ketoglutarate dehydrogenase (KGDH), succinate dehydrogenase (SDH), and malate dehydrogenase (MDH) activity was carried out as previously described [18]. *V. alginolyticus* cultured in medium with Ala or/and Gent were collected and washed three times with 1× PBS (pH 7.0). The bacterial cells were suspended in 1× PBS (pH 7.0) and adjusted to OD_600_ nm at 1.0. Aliquot of 30-mL cells were collected and transferred to a 1.5-mL centrifuge tube. The cells were resuspended with 0.6 mL 1× PBS and disrupted by sonic oscillation for 6 min (200 W total power with 35% output, 2 s pulse, 3 s pause over ice). Following by centrifugation at 12,000 × g rpm for 10 min at 4°C, supernatants were collected. Protein concentration of the supernatant was quantified by BCA protein concentration determination kit (Beyotime, P0009). Then, 200 μg proteins were used for determination of enzyme activity. The same proteins were also used to measurement of NOS activity with reference to the nitric oxide synthase typed assay kit by colorimetric method (Nanjing Jiancheng Bioengineering Institute, A014-1). In detail, 50 μL of 4 mg/mL proteins were mixed with 100 μL water, 200 μL substrate buffer, 10 μL reaction promoter, and 100 μL color reagent at 37°C for 15 min. The reaction solution was added transparent agent and terminator and then mixed well. The absorbance was measured at 530 nm with a cuvette with 1 cm optical path and the same volume of protein buffer was used as a blank control group. When L-NMMA was used, 100-μg proteins were mixed with 0.5 mM L-NMMA at 30°C for 2 h before the measurement. tNOS activity (U/mg) was calculated as following formula: tNOS activity = (Experimental group OD value – blank OD value)/fix coefficient × (reaction solution volume/protein volume × (1/(colorimetric path × reaction time)/protein concentration.

### NO quantification

The measurement was performed using the same samples as above for the enzyme activity determination. NO quantification was completed according to the total NO assay kit by nitrate reductase method (Nanjing Jiancheng Bioengineering Institute, A012-1). In detail, 500 μL of 4 mg/mL proteins are mixed with 400 μL buffer I at 37°C for 60 min. The reaction solution was added 300 μL buffer II and vortexed and mixed well for 30 s and then sedimented at 25°C for 40 min. Supernatant was obtained by concentration at 1,600 × g for 10 min. Aliquot of 800 μL supernatant was mixed with 600 μL color developer and sedimented at 25°C for 10 min. Absorbance was measured at 550 nm with a cuvette with 0.5 cm optical path. The same volume of protein buffer and 0.1 mmol/L standard sample were used as a blank control and a reference for concentration calculation, respectively. NO concentration (μmol/g) was calculated as following formula: NO concentration = (Experiment group OD value – blank OD value)/(standard sample OD value – blank OD value) × standard sample concentration/protein concentration.

### Nitrite quantification

The measurement was performed using the same samples as above for the enzyme activity determination. Nitrite quantification was performed using commercial kit (Nanjing Jiancheng Bioengineering Institute, A038-1-1). In brief, the bacterial cells were suspended in saline and adjusted to OD_600_ nm at 1.0. Aliquot of 60 mL cells were collected and transferred to a 1.5-mL centrifuge tube. The cells were resuspended with 0.8-mL saline and disrupted by sonic oscillation for 10 min (200 W total power with 35% output, 2 s pulse, 3 s pause over ice). Aliquot of 800 μL (10 mg/mL) proteins were reacted with 1,200 μL buffer reagent at 25°C for 10 min. Supernatant was obtained at 1600 × g for 10 min. Aliquot of 800 μL supernatant and 400 μL color developer were mixed and sedimented at 25°C for 15 min. Absorbance was measured at 550 nm with a cuvette with 0.5 cm optical path. The same volume of saline was used as a blank control group, 100 μmol/L standard samples were used as a reference for concentration calculation. Finally, nitrite content was calculated as following formula, nitrite concentration unit is defined as μmol/L: nitrite content = (experimental group OD value – blank OD value)/(Standard sample OD value – blank OD value) × standars sample content × protein sample dilution factor.

### Blue native PAGE

Oligomeric state of rCysJ was analyzed by BeyoGel™ Plus PAGE 4–20% Bis-Tris precast gels (beyotime, P0524S). Briefly, equal volumes of 0.5 or 1.0 μg/mL rCysJ with 2 × BN-PAGE loading buffer (Sangon Biotech Co., Ltd., Shanghai) were mixed. Anode buffer A (25 mM imidazole, pH 7.0) was added to the inner tank and cathode buffer B (7.5 mM imidazole, 50 mM tricine, 0.02% coomassie blue G-250, pH 7.0) was added to outer tank. Electrophoresis was carried out at a voltage of 100 V constant voltage for 20 min. When protein entered the concentrated gel, electrophoresis was performed at a voltage of 120 V constant voltage for 30 min. Then, the electrophoresis buffer cathode buffer B was replaced with cathode buffer B/10 (7.5 mM imidazole, 50 mM tricine, 0.002% coomassie blue G-250, pH 7.0). The electrophoresis continued at 120 V constant voltage until prestained protein ladder (TSINGKE, TSP021) indicator strip is near the bottom of the electrophoresis.

### *Δ*cys*Jconstruction*

A genetic deletion mutant was constructed via homologous recombination procedures using the pDS132 suicide vector system as previously described [19,20]. Briefly, using the genomic DNA of *V. alginolyticus* ATCC33787 strain as a template, DNA segments with about 500 bp that were located upstream (*cysJ*-P1/ *cysJ*-P2) and downstream (*cysJ*-P3/ *cysJ*-P4) of *cysJ* were amplified by PCR (Fig. S5A). The PCR products were recombined with the pDS132 plasmid by overlapping cloning with ClonExpress^R^ Kit (Vazyme Biotech Co., Ltd., Nanjing, China). The constructed vector was sequentially transferred into *E. coli* MC1061 to increase the transformation efficiency and quickly propagate it, and then transferred to *E. coli* MFD-λpir (survived in medium containing 2,6-Diaminopimelic acid). Both transformations were plated on LB agar plate with chloramphenicol and positive colonies were verified by PCR (Figs. S5B and S5C). Correctly recombined *E. coli* MFD-λpir strains were conjugated with *V. alginolyticus* by mixing both strains at 1:4 ratios and spotted on a sterile nitrocellulose filter in LB agar at 30°C overnight for the first homologous recombination. The mixture was washed with fresh LB medium and spread on LB agar plate containing ampicillin (100 µg/mL) and chloramphenicol (25 µg/mL). The positive colonies were picked up and cultured in LB medium overnight and then spotted on LB agar plate containing 20% sucrose or 25 µg/mL chloramphenicol. The colonies that grew on the sucrose LB agar plate but were sensitive to chloramphenicol were further verified by PCR (Figs. S5D and S5E) and sequencing. The primer sequences used in this study are provided in Table S2.

### Quantitative real-time polymerase chain reaction

Quantitative real-time polymerase chain reaction (qRT-PCR) was performed as previously described [8]. In brief, overnight bacteria were collected and wash three times with M9 medium. Then, the bacteria were diluted in M9 medium to arrive at 0.6 of OD_600_ nm and dispensed into tubes, 5 mL each tube, with or without desired metabolites, L-NMMA or/and antibiotics. Following by shaking at 30°C for 6 h, cells were collected and adjusted 1.0 of OD_600_ nm. Aliquot of 1 mL was transferred to a 1.5 mL QSP centrifuge tube. Supernatant was removed by centrifugation and pellet was used for total RNA isolation using TRIZOL reagent (Invitrogen Life Technologies). Total RNA 1 μg as template was used for qRT-PCR by a PrimeScript RT reagent kit with gDNA Eraser (Takara, Japan) according to manufacturer’s instructions. The primers used for qRT-PCR are shown in Table S1 (*cysJ*-F2 and *cysJ*-R2), where the 16S rDNA gene was served as an internal control. qRT-PCR was performed in 384-well plates with a total volume of 10 μL. The reaction mixtures were run on a LightCycler 480 system (Roche, Germany). The cycling parameters were listed as follows: 95°C for 30s to activate the polymerase, 50 cycles of 95°C for 5s, 58°C for 30s. Fluorescence measurements were performed at 75°C for 1°s during each cycle. Cycling was terminated at 95°C with a calefactive velocity of 0.11°C s^−1^ to obtain a melting curve. Data are shown as relative mRNA expression compared with the control group with the endogenous reference 16S rRNA gene.

## Results

### Gent-mediated killing promoted by Ala

**Figure 1.**
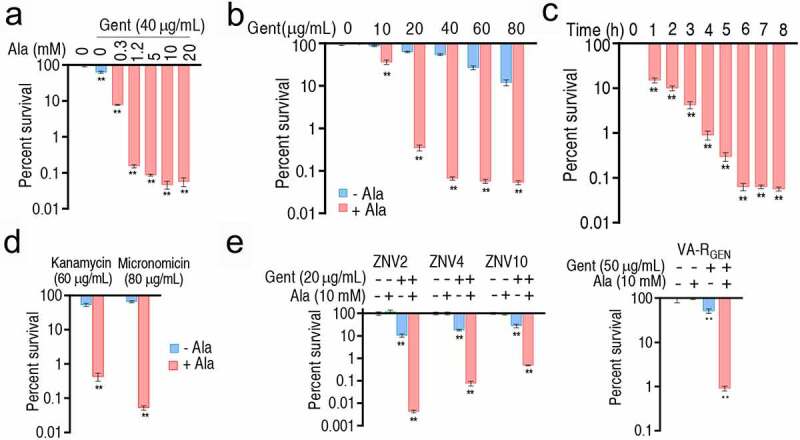
Antibacterial efficiency by the synergistic use of Gent and Ala. A. Percent survival of ATCC33787 in the indicated concentrations of Ala and 40 μg/mL Gent. B. Percent survival of ATCC33787 in the indicated concentrations of Gent and 10 mM Ala. C. Percent survival of ATCC33787 in the indicated incubation time in the synergistic use of 40 μg/mL Gent and 10 mM Ala. D. Percent survival of ATCC33787 in the presence of other aminoglycosides. E. Percent survival of clinically isolated *Vibrio* spp. and lab-evolved antibiotic-resistant strains in the synergistic use of the indicated concentrations of Gent and 10 mM Ala. Results (a-e) are displayed as mean ± SEM, and significant differences are identified **p < 0.01) as determined by two-tailed Student’s t test

To carefully examine effect of exogenous Ala on Gent-mediated killing to *V. alginolyticus*, viability of ATCC33787 was detected in the presence of different concentrations of exogenous Ala plus 40 μg/mL Gent. A dose-dependent killing was determined, where 10 mM Ala led to the most synergistic effect of killing efficacy (Figure 1a). Then, different concentrations of Gent with 10 mM exogenous Ala were used. Percent survival of ATCC33787 was reduced with the increasing doses of Gent and arrived at the top of the killing efficacy in 40 μg/mL Gent by 815.9-fold (Figure 1b). Finally, suitable incubation period of the synergistic use of Gent and Ala was tested. The most killing efficacy was found at 6–8 h (Figure 1c). In addition, the Ala-potentiated efficacy was detected on using other aminoglycosides and clinically isolated and lab-evolved antibiotic-resistant *V. alginolyticus* strains. Specifically, Ala-elevated kanamycin and micronomicin to kill ATCC33787 by 125- and 1,257-folds, respectively (Figure 1d). Exogenous Ala promoted the Gent-mediated killing to these clinically isolated and lab-evolved antibiotic-resistant strains (Figure 1e). These results indicate that the synergistic use of exogenous Ala and aminoglycosides is effective to kill lab-evolved and clinically isolated antibiotic-resistant *V. alginolyticus* strains.

### Metabolic shifts induced by Ala, Gent, and both

**Figure 2.**
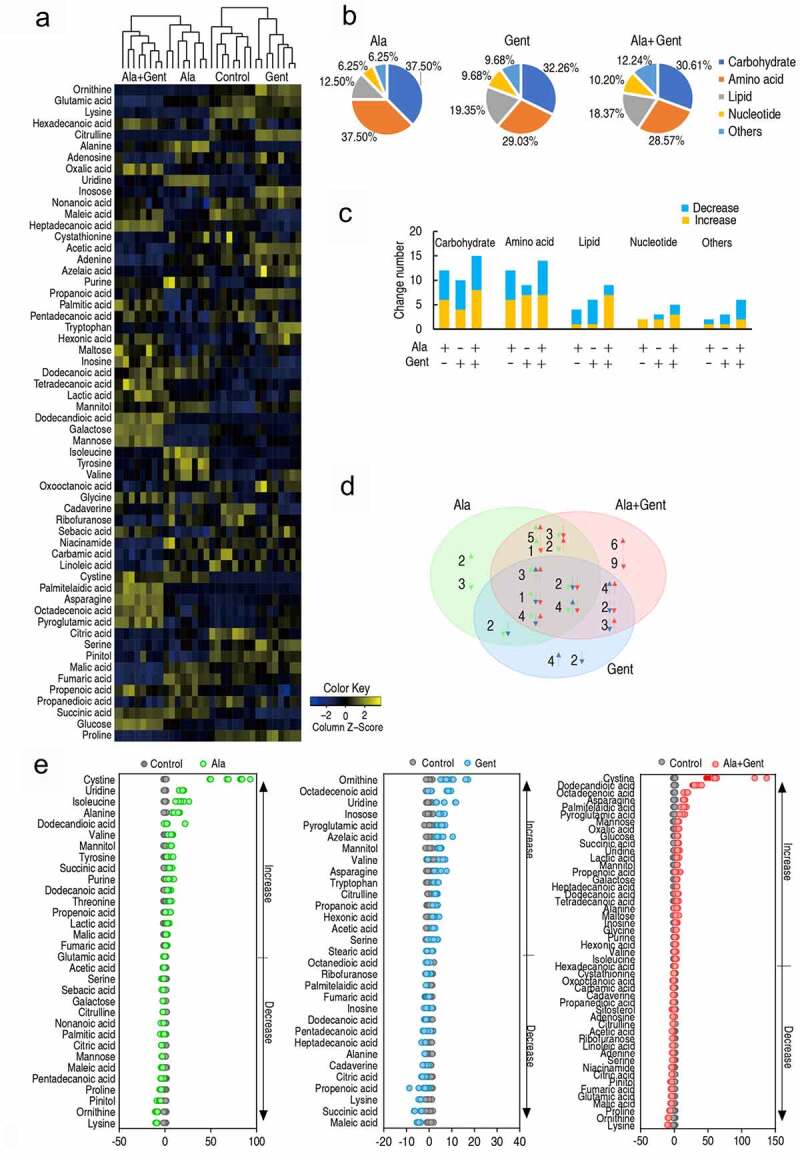
Differential metabolomic profiling of Ala group, Gent group, and Ala + Gent group compared with untreated group (control). A. Heat map showing differential metabolites. Yellow color and blue color indicate increase and decrease of metabolites relative to the median metabolite level, respectively (see color scale). B. Category of identified metabolites of differential abundance. C. Number of differential abundance of metabolites. D. Venn diagram for comparison of differential metabolites among Ala (green color), Gent (blue color) and Ala + Gent group (red color). E. Z-score plot of differential metabolites based on control. The data of Ala (left), Gent (middle) and Ala + Gent (right) groups were separately scaled to the mean and standard deviation of control. Each point represents one metabolite in one technical repeat and colored by sample types

To understand metabolic mechanisms by which exogenous Ala potentiates the Gent-mediated killing, GC-MS-based metabolomics was used to compare the metabolic shifts of ATCC33787 in the presence of exogenous Ala, Gent and both. Four biological samples with two technical replicas each group yielded 32 data sets. Total 74 metabolites were identified in each sample and categorized into carbohydrate (25.68%), amino acid (29.73%), lipid (21.62%), nucleotide (10.81%), and other (12.16%). Cluster analysis showed that the four groups are clearly separated. Control group and Gent group are closely linked, while Ala + Gent group is closely linked with Ala group (Figure S1). A Kruskal–Wallis test (p < 0.05) was used to identify differential abundance of metabolites. Compared with control group, a total of 58 metabolites were determined, where 32, 31, and 49 differentially abundant metabolites were found in Ala group, Gent group and Ala + Gent group, respectively (Figure 2a). Among these metabolites, carbohydrate (37.5%, 32.26%, and 30.61%), amino acid (37.50%, 29.03%, and 28.57%), fatty acid (12.5%, 19.35%, and 18.37%), nucleotide (6.25%, 9.68%, and 10.20%), and other metabolites (6.25%, 9.68%, and 12.24%) were detected in the three groups (Ala group, Gent group and Ala + Gent group, respectively) (Figure 2b). Number and distribution of these differential metabolites at abundance were shown in (Figure 2c). Among these differential metabolites, 14 were overlapped; 2, 11, and 9 were shared between Ala group and Gent group, Ala group and Ala + Gent group, Gent group and Ala + Gent group, respectively; 5, 6, and 15 existed only in Ala group, Gent group and Ala + Gent group, respectively (Figure 2d). Z-score varied between −9.28 and 72.47 in Ala group, −4.60 and 10.12 in Gent group and −8.74 and 74.70 in Ala + Gent group (Figure 2e). These results indicate that Ala, Gent, and both induce differential metabolomes, where more changes are determined in Ala + Gent-induced metabolome than the other metabolomes.

### Enriched pathways impacted by Ala, Gent, and both

**Figure 3.**
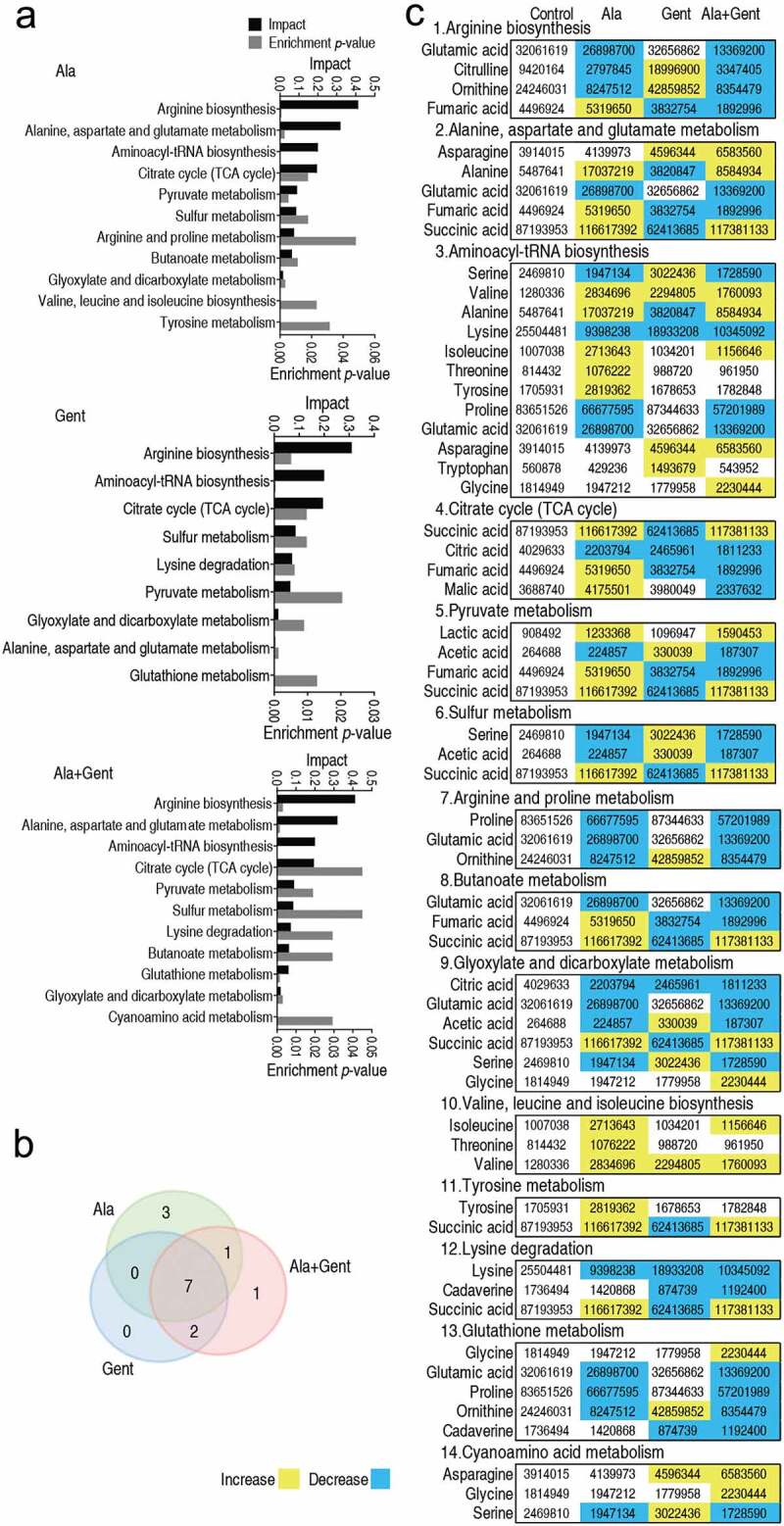
Pathway enrichment. A. Pathway enrichment of varied metabolites in the Ala group, Gent group, and Ala + Gent group. Significant enriched pathways are selected to plot. *P*-value < 0.05. B. Venn diagram for significantly enriched pathways between the Ala (green color), Gent (blue color), and Ala + Gent group (red color). C. Integrative analysis of metabolites in significantly enriched pathways. Yellow color and blue color indicate increased and decreased metabolites, respectively

Metabolic pathways are a linked series of chemical reactions, leading to anabolism or breakdown of metabolites within a cell. Thus, investigation of differential metabolic pathways is key to understand the differential abundance of metabolomes induced by exogenous Ala, Gent, and both. When the differential abundance of metabolites were separately analyzed, 11, 9, and 11 metabolic pathways were enriched in Ala group, Gent group, and Ala + Gent group, respectively (Figure 3a). Among them, seven metabolic pathways (arginine biosynthesis; alanine, aspartate and glutamate metabolism; aminoacyl-tRNA biosynthesis; citrate cycle; pyruvate metabolism; sulfur metabolism; arginine and proline metabolism; butanoate metabolism, and glyoxylate and dicarboxylate metabolism) were shared in the three groups, while butanoate metabolism, lysine degradation, and glutathione metabolism were overlapped, respectively, in Ala group and Ala + Gent group and in Gent group and Ala + Gent group (Figure 3b). Interestingly, Arg biosynthesis was the most impacted pathway in the three groups, where four metabolites were detected. Among them, the decreased glutamic acid, citrulline, ornithine and the elevated fumaric acid in Ala group were found, while reversal change, the elevated citrulline and ornithine and the reduced fumaric acid, was detected in Gent group. However, the four metabolites were reduced in Ala + Gent group (Figure 3c), indicating effect of the synergistic use of Ala and Gent that is different from that caused by Ala or Gent alone. Arg biosynthesis contributes to NO production, which is related to antibiotic resistance [21,22]. Thus, the most impacted Arg biosynthesis is an interesting clue to explore the mechanisms by which Ala potentiates Gent to kill *V. alginolyticus*.

### Biomarkers induced by Ala, Gent, and both

**Figure 4.**
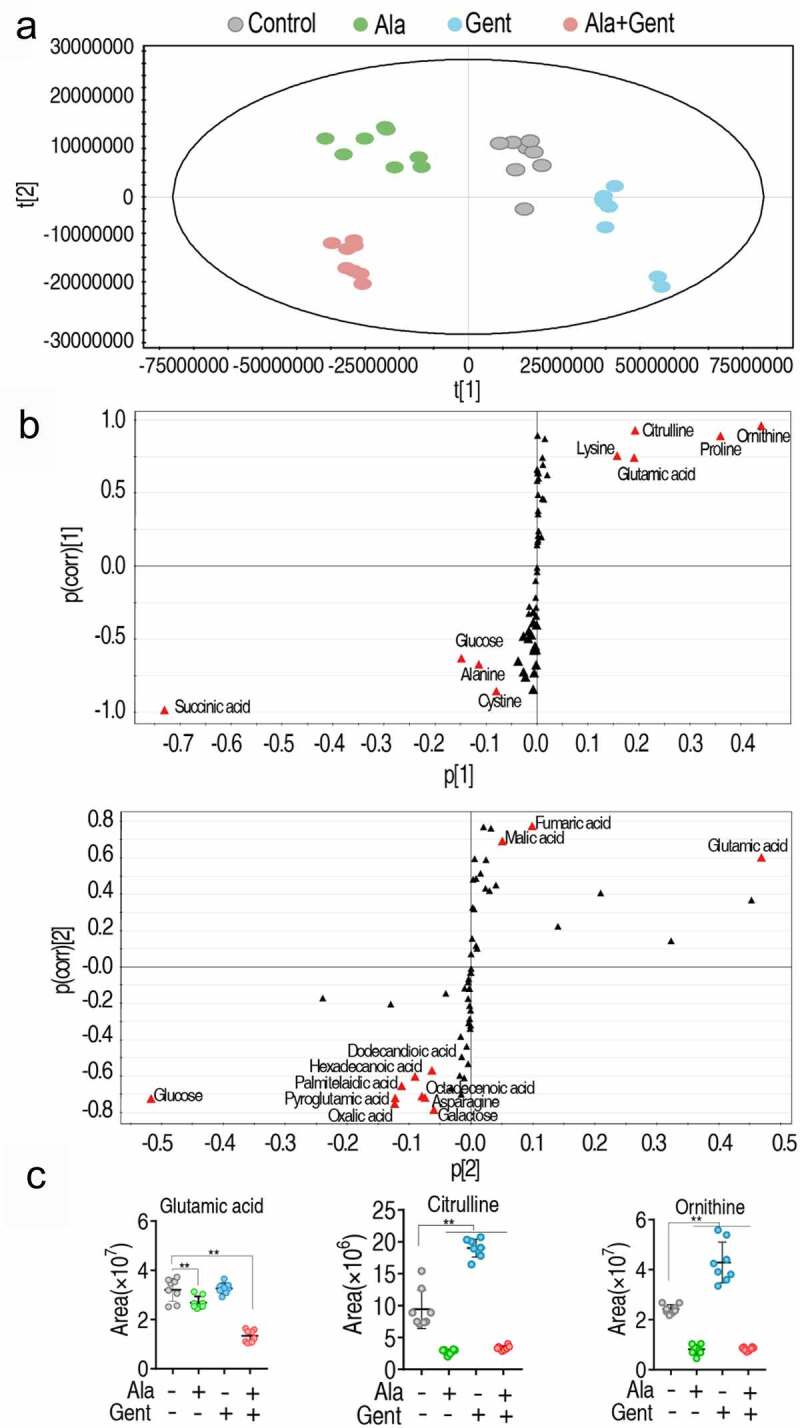
Identification of crucial metabolites. A. The PCA analysis of control group, Ala group, Gent group, and Ala + Gent group. Each dot represents the technique replicates in the plot. t [1] and t [2] explain 98.7% of the total variance which allows confident interpretation of the variation. B. S-plot generated from OPLS-DA. Triangle represents individual metabolite, where potential biomarkers are highlighted with red, which is greater or equal to 0.05 and 0.5 for absolute value of covariance p and correlation p(corr), respectively. C. The scatter plot of glutamic acid, citrulline, and ornithine. Results are displayed as mean ± SEM, and significant differences are identified **p < 0.01

Pattern recognition method is an efficient tool to identify biomarkers in metabolomics analysis. Thus, orthogonal partial least square discriminant analysis (PLS-DA) was carried out to recognize the sample patterns of metabolomes as showed in (Figure 4a). Component t [1] separated Gent group and control group from Ala group and Ala + Gent group, while component t [2] differentiated Ala group and control group from Gent group and Ala + Gent group. Furthermore, discriminating variables were shown by S-plot. In the plots of predictive correlation between p [1] and p(corr) [1] and p [2] and p(corr) [2], the red triangle indicates the differential metabolites that have larger weights (< −0.05 or > 0.05) and higher relevance (< −0.5 or > 0.5). The analysis led to the identification of 9 (ornithine, proline, citrulline, glutamic acid, lysine, Ala, glucose, cysteine and succinic acid) and 12 (glutamic acid, fumaric acid, malic acid, dodecandioic acid, octadecenoic acid, asparagine, galactose, hexadecanoic acid, palmitelaidic acid, pyroglutamic acid, oxalic acid and glucose) biomarkers in the correlation between p [1] and p(corr) [1] and p [2] and p(corr) [2], respectively (Figure 4b). Among them, glutamic acid, citrulline, and ornithine belong to Arg biosynthesis, the most impacted pathway. Interestingly, abundance of the three biomarkers was differential compared to control and different among the three groups. Specifically, abundance of glutamic acid was no difference between in Ala group and Gent group but low in Ala + Gent group; Abundance of citrulline and ornithine was decreased in Ala group and Ala + Gent group but elevated in Gent group as described earlier (Figure 4c, Figure S2). Thus, the synergistic use of Ala + Gent reduces abundance of the three biomarkers belonging to Arg biosynthesis, while Ala or Gent alone only decreases one or two out of them.

### Metabolic fluxes impacted by Gent, Ala, and both

**Figure 5.**
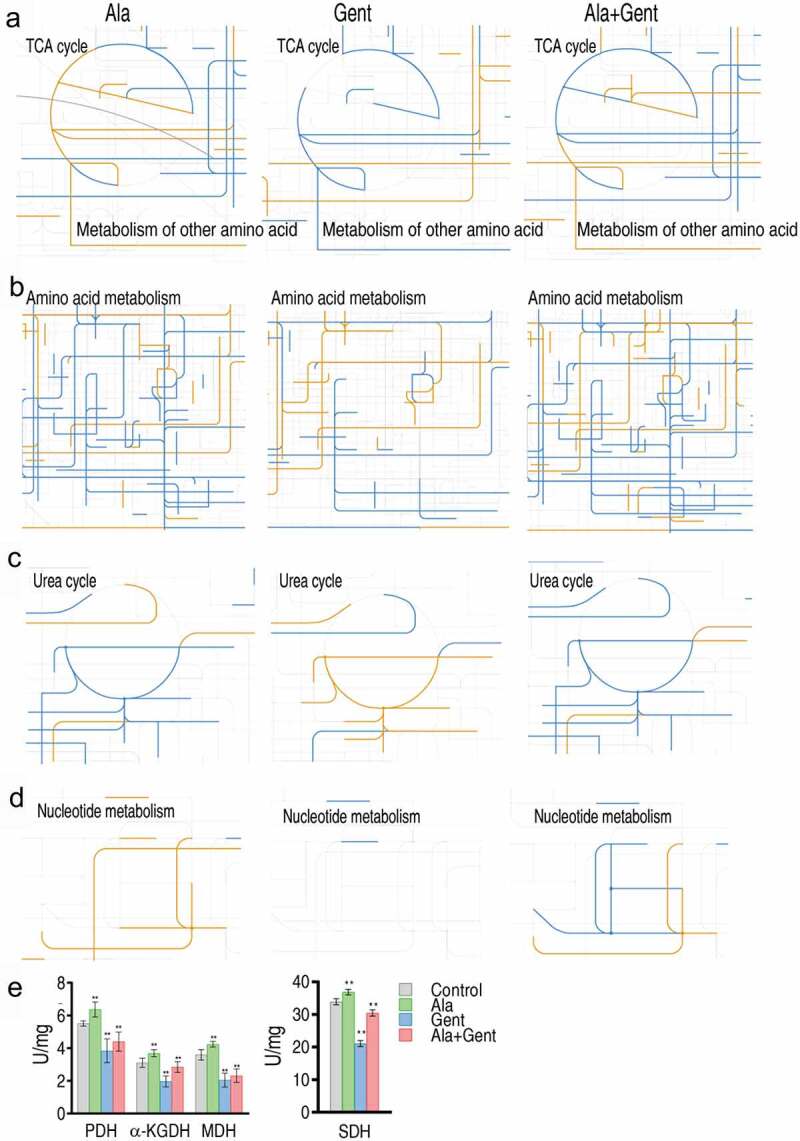
iPath analysis showing comparison among Ala group, Gent group, and Ala + Gent group. The yellow and blue lines mean upregulation and downregulation of metabolic pathways, respectively. A. The TCA cycle. B. Amino acid metabolism. C. Urea cycle. D. Nucleotide metabolism. E. Activity of enzymes of the P cycle. Results (e) are displayed as mean ± SEM, and significant differences are identified (**p < 0.01) as determined by two-tailed Student’s t test

To understand the global metabolic fluxes of ATCC33787 under exogenous Ala, Gent, and both, data on differential abundance of metabolites had been submitted for analysis through the online interactive iPath3.0 (Figure S3). It can be seen that the resulting global overview maps are different among the three groups, suggesting that the cooperative effect between Ala and Gent plays a role. Specifically, fluctuation of the TCA cycle in Ala + Gent group is attributed to an affect caused by Ala and Gent, where Gent played a greater role than Ala (Figure 5a). Flux of amino acid metabolism in Ala + Gent group resulted from the integration of Ala group and Gent group, where Ala-induced metabolism was predominant (Figure 5b). Combination of Ala- and Gent-mediated urea cycle and NO pathway resulted in more depression of the cycle and pathway (Figure 5c). While Ala promoted and Gent weakened nucleotide metabolism, the synergistic use led to fluctuation of nucleotide metabolism (Figure 5d). Meanwhile, activity of pyruvate dehydrogenase (PDH), α-ketoglutarate dehydrogenase (KGDH), succinate dehydrogenase (SDH), and malate dehydrogenase (MDH) was measured. Ala and Gent promoted and inhibited the activity of all enzymes, respectively, while the synergy of Ala and Gent caused decrease of PDH, SDH and MDH activity (Figure 5e). The analysis is generally consistent with the data in (Figure 5(a,d)). Since the TCA cycle provides source for nucleotide metabolism via glutamate metabolism, the results further support the idea that NO production pathway can be used to explore the metabolic mechanisms by which Ala potentiates Gent-mediated killing.

### NO decrease mediated by Gent, Ala and both

**Figure 6.**
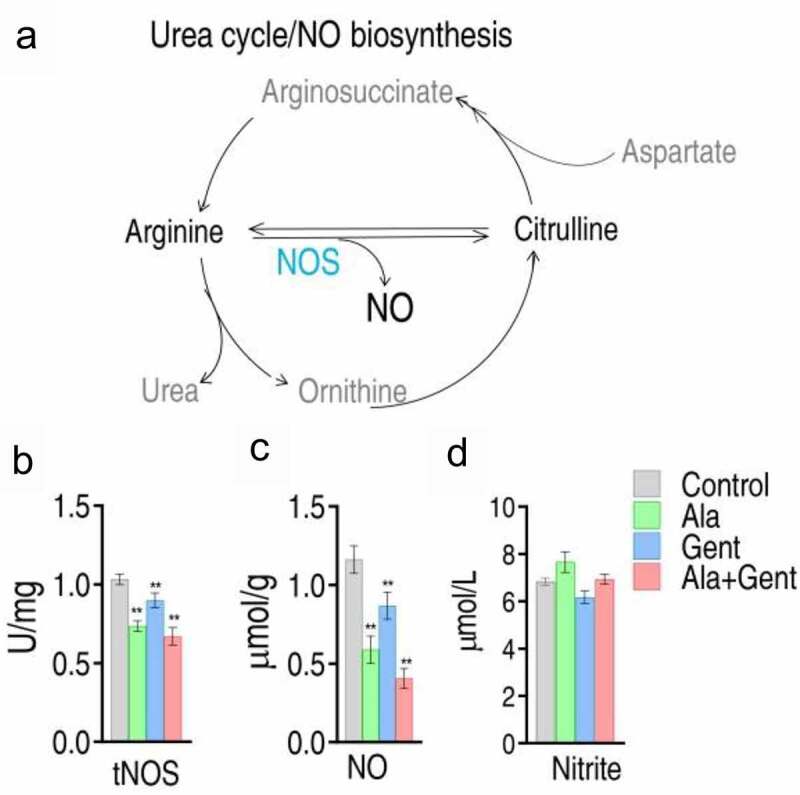
NO generation and level mediated by Gent, Ala, and both. A. Metabolic diagram of NO generation. B. Activity of NO synthase (NOS) in *V. alginolyticus* ATCC33787 in response to Ala, Gent, and both. C. NO level in *V. alginolyticus* ATCC33787 in response to Ala, Gent, and both. D, Nitrite in *V. alginolyticus* ATCC33787 in response to Ala, Gent, and both. Results (b and c) are displayed as mean ± SEM, and significant differences are identified (*p < 0.05, **p < 0.01) as determined by two-tailed Student’s t test

NO generation occurs through L-Arg, a reaction catalyzed by NOS, to produce NO and L-citrulline (Figure 6a). To demonstrate that NO formation is reduced in the synergistic use of Ala and Gent, activity of NOS and level of NO was measured in ATCC33787 with the three treatments. These treatments reduced activity of total NOS (tNOS), where the activity was ranked from high to low: Gent group > Ala group > Ala + Gent group (Figure 6b). Consistently, change in NO level was the same as that of tNOS in the three treatments (Figure 6c). These results are consistent with metabolomic analysis that the most reduced NO is detected in the synergistic use of Ala and Gent. Thus, the decrease of NO forms a characteristic feature in the Ala-enabled killing of *V. alginolyticus* by aminoglycosides. In addition, to exclude the role of nitrite-dependent NO biosynthesis in the NO decrease, we showed that Ala, Gent and both do not change level of nitrite (Figure 6d), which is the substrate of nitrite oxidoreductase in nitrite-dependent NO biosynthesis. The data support the conclusion that Alan, Gent and both inhibit NO via arginine-dependent NO biosynthesis.

### NO decrease contributes to the Ala-potentiated Gent killing

**Figure 7.**
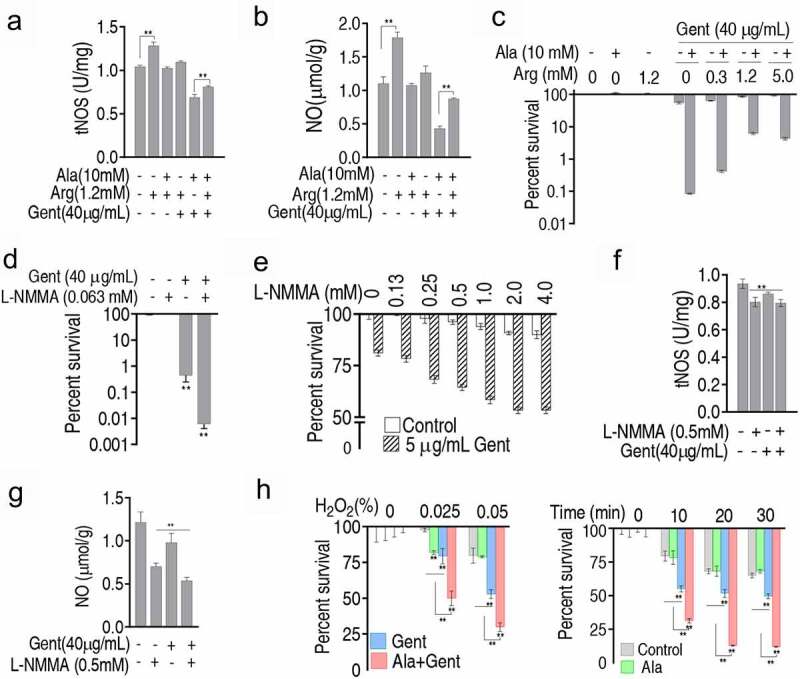
Association of NO level with percent survival. A and B. NOS activity (a) and NO level (b) of *V. alginolyticus* ATCC33787 in the absence or presence of Ala, Gent or/and arginine. C. Percent survival of *V. alginolyticus* ATCC33787 in the presence of Ala + Gent plus the indicated concentration of arginine. D. Percent survival of *V. alginolyticus* ATCC33787 in the presence of Gent and L-NMMA. E. Percent survival of *V. alginolyticus* ATCC33787 in the presence of Gent plus the indicated concentration of L-NMMA. F. and G. NOS (f) and NO (g) of *V. alginolyticus* ATCC33787 in the presence of Ala + Gent plus L-NMMA. H. Percent survival of *V. alginolyticus* ATCC33787 in the presence of Ala and Gent, and the indicated concentration of H_2_O_2_ and time. Results (a-h) are displayed as mean ± SEM, and significant differences are identified (*p < 0.05, **p < 0.01) as determined by two-tailed Student’s t test

To explore whether the NO decrease plays a role in the Ala-potentiated Gent killing, L-Arg was used since the amino acid promotes NO formation. L-Arg promoted activity of tNOS and production of NO, which was blocked by Ala or Gent. The synergistic use of Ala + Gent led to lower activity of tNOS and product of NO than control, which was partly recovered by L-Arg (Figure 7(a,b)). Consistently, L-Arg elevated the survival of *V. alginolyticus* ATCC33787 caused by Ala + Gent with increasing dose of L-Arg (Figure 7c). On contrary, NOS inhibitor N^G^-Monomethyl-L-arginine (L-NMMA) promoted Gent-mediated killing to *V. alginolyticus* ATCC33787 (Figure 7d) and in an L-NMMA dose-dependent manner (Figure 7e). This is consistent with these results that activity of tNOS was inhibited by L-NMMA (figure 7f). NO level exhibited as tNOS did in the three treatments (Figure 7g). Reports have shown that endogenous NO production protects bacteria against H_2_O_2_-mediated killing [23]. Bacteria that survived following treatment with the synergistic use of alanine and gentamicin were sensitive to H_2_O_2_-mediated killing (Figure 7h). These results indicate that the decrease of NO induced by the synergistic use of Ala and Gent plays a role in the killing.

### *Identification of NOS gene in* V. alginolyticus *ATCC33787*

**Figure 8.**
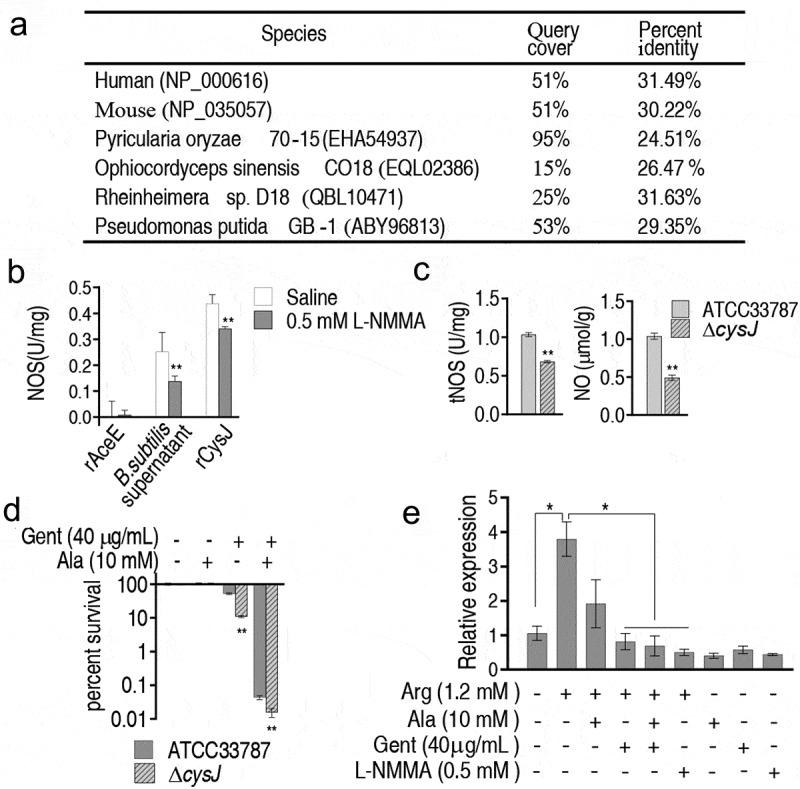
**Role of** CysJ **in NO generation in** *V. alginolyticus*. A. Blast analysis of NOS-like protein CysJ in *V. alginolyticus* against NOS from human, mouse, fungi, and bacteria. B. Activity of NOS in recombinant CysJ in the absence or presence of SDS or L-NMMA. C. Activity of NOS and content of NO in *cysJ*-deleted mutant. D. Percent survival of *cysJ*-deleted mutant. E. qRT-PCR for expression of *cysJ* in the presence of Ala, Gent, L-NMMA or/and arginine

Whether ATCC33787 has NOS and which enzyme plays in a role have not been reported. Thus, homology analysis of NOS was performed against ATCC33787 genome. CysJ (AT730_09320) was identified as the most homologous protein against NOS from human, mouse, fungi and bacteria (Figure 8a). Gene encoding CysJ was cloned into pET-32a vector which was used as expression vector in *E. coli* BL21 (Figure S4A). A band was detected in the corresponding location of recombinant CysJ (rCysJ) using SDS/PAGE (Figure S4B). A dimer of molecular mass of rCysJ was identified by PAGE (Figure S4C). Activity of the purified rCysJ was measured when purified rAceE and supernatant from *Bacillus subtilis* were used as negative and positive controls, respectively. The activity was detected in rCysJ and the positive control but not in the negative control. When L-NMMA was used, activity of NOS was reduced (Figure 8b). Moreover, a *cysJ*-deleted mutant was constructed (Figure S5). Absence of *cysJ* inhibited activity of ROS and reduced level of NO (Figure 8c). Consistently, viability was lower in *cysJ*-deleted mutant than control (Figure 8d). Finally, qRT-PCR showed that L-Arg promoted expression of *cysJ*, which was partly and completely inhibited by Ala and Gent (with Ala) or L-NMMA, respectively (Figure 8e). These results indicate that CysJ plays a role in endogenous NO generation of ATCC33787.

## Discussion

Cell metabolic states contribute to functional responsibilities [4,5,24–30]. When antibiotic-resistant metabolomes are reprogrammed to antibiotic-sensitive metabolomes using crucial biomarkers identified from differential metabolomes between the antibiotic-resistant and -sensitive metabolomes, bacteria become sensitive to the existing antibiotic-mediated killing [5–10]. Since the crucial biomarkers and antibiotics could separately lead to metabolic shifts [15,16,31], the reprogrammed metabolomes sensitive to antibiotics should be resulted from a synergy of both crucial biomarker and antibiotics. However, the crucial biomarkers-induced metabolomes have been characterized [5,10], but the synergistic effect of both crucial biomarkers and antibiotics on the reprogrammed metabolomes is unknown. The present study explores the cooperative effect on metabolic mechanisms by which Ala promotes Gent-mediated killing. Our cluster analysis showed that control group and Gent group are closely linked, while Ala group is closely linked with Ala group + Gent. Exogenous Ala dominates the metabolic profile, but Gent also plays a role, indicating that the synergistic effect contributes to the reprogrammed metabolome. Our previous report showed that glucose and Ala potentiate antibiotic-mediated killing, but the killing efficacy is differential in different classes of antibiotics, where the efficacy from high to low ranks aminoglycoside (Gent) > quinolones (balofloxacin) > β-lactam (ceftazidime and ampicillin) [5]. Thus, the present findings are useful to explain why the metabolites-enabled killing of antibiotic-resistant bacteria by antibiotics is antibiotic classes-dependent. This is because the elevated killing efficacy should be attributed to the synergistic effect of both metabolites and antibiotics on the reprogrammed metabolomes.

Among the altered metabolic states, the most impacted pathway arginine biosynthesis and crucial metabolites, glutamate, citrulline, and ornithine, are related to NO production pathway [32]. Unfortunately, the present study failed in identification of arginine, which is a substrate catalyzed by NOS. Ala and Gent differentially modulated the metabolic pathway. Specifically, exogenous Ala and Gent caused metabolites of the pathway to be mostly reduced and elevated, respectively, whereas the synergistic use of Ala and Gent made all detected metabolites reduced. The difference is linked to the differentially Gent-mediated killing efficacy with and without exogenous Ala, i. e. lower and higher killing were detected in Gent group and Ala + Gent group, respectively. Thus, the present study used the clue to explore metabolic modulation on the Ala-potentiated Gent-mediated killing. Our results demonstrate that NO decrease plays a role in Ala-potentiated Gent-mediated killing. The decrease is caused by not only exogenous Ala but also Gent. Specifically, NO production is related to urea cycle directly and the P cycle indirectly (providing aspartate for urea cycle), which were affected by exogenous Ala and Gent in the Ala + Gent group. These results indicate that both reprogramming metabolite Ala and antibiotic Gent play a role in the reprogrammed metabolome. NO-mediated resistance to aminoglycosides is not reported in *V. alginolyticus*, but NO protects other bacteria including Gram-negative *Escherichia coli, Salmonella enterica*, and *Pseudomonas aeruginosa* and the Gram-positive *Staphylococcus aureus* from killing mediated by aminoglycosides [33,34]. This is because blockage of aerobic respiration by high NO fluxes reduces drug uptake, thereby promoting aminoglycoside resistance [33].

Gene expressing NOS, catalyzing NO production from L-arginine, is not defined in *V. alginolyticus*, but the present study utilized commercial kits to show NOS activity and NO content and their changes with metabolites and antibiotic. Furthermore, the activity of NOS was promoted and inhibited by arginine and L-NMMA, respectively. These results suggest a NOS-like protein in the bacterium. Several species of bacteria possess NOS that oxidizes L-arginine to produce low concentrations of NO [35,36]. NOS-like proteins have been identified in many bacteria *Deinococcus radiodurans, Bacillus subtilis, Bacillus halodurans, Bacillus anthracis*, and *Staphylococcus aureus* [37,38], but not in *Vibrio* spp. To explore which protein plays a role as NOD-like protein does in *V. alginolyticus*, homology analysis of NOS was performed against ATCC33787 genome and CysJ (AT730_09320) was selected. Recombinant CysJ showed the NOS activity. Absence of *cysJ* caused loss of the activity and elevated sensitivity to Gent and the synergistic use of Ala and Gent. Expression of *cysJ* was regulated by Ala, Gent, L-NMMA, and Arg. The present study indicates that NOS and NO exist in *V. alginolyticus* and CysJ is a NOS-like protein in this bacterium.

Compared with control group without Ala and Gent, Gent or/and Ala caused low NOS activity and NO content, ranking from low to lowest as a result of Gent > Ala > both. The reduced NOS activity and NO content contributed to Ala-potentiated Gent-mediated killing as demonstrated by the following experiments. First, NO-precursor arginine promoted bacterial viability in a dose-dependent manner. Second, NO inhibitor inhibited bacterial viability with the increasing dose. Third, survivor cells from alanine-potentiated gentamicin-mediated killing are sensitive to H2O2. Fourth, absence of *cysJ* led to increasing sensitivity to the killing. These results support the conclusion that the synergistic effect of Ala and Gent on the reduced NO contributes to Ala-potentiated Gent-mediated killing. A line of evidence has indicated that NO level is related to bacterial sensitivity or resistance to antibiotics [39,40]. The finding on the role of NO in impacting Gent-mediated killing is not new [34], but the NO decrease in this case is attributed to the synergy of Ala and Gent and this plays a role are novel.

**Figure 9.**
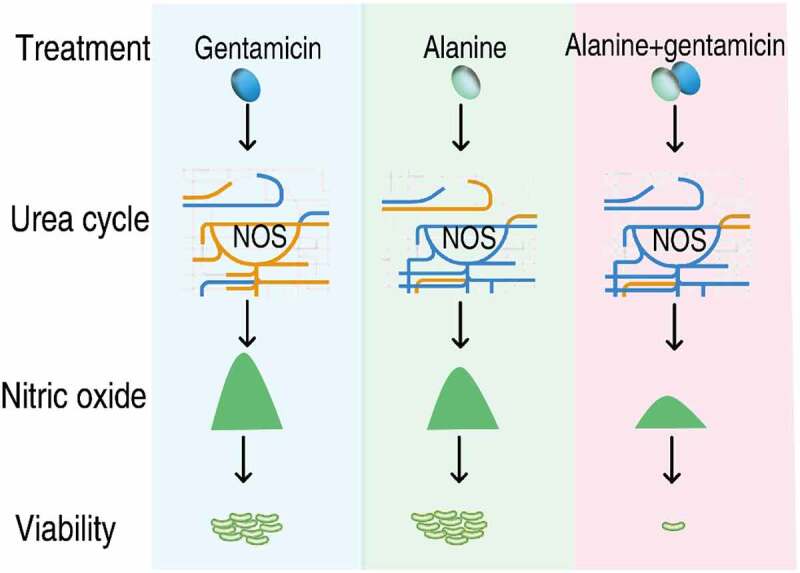
Diagram for the synergistic use of Gent and Ala to kill antibiotic-resistant *V. alginolyticus.*

In summary, the present study explores the cooperative effect of exogenous Ala and Gent on effective killing to *V. alginolyticus*. Metabolomic analysis identifies NO production pathway as a key clue to understand the effect. Exogenous Ala and Gent inhibited NO production, but the combination of Ala and Gent caused lower NO production than Ala or Gent alone. The decrease of NO level contributed to the Ala-potentiated Gent-mediated killing (Figure 9). These findings indicate that both metabolome-reprogramming metabolite and antibiotic play a role in the reprogramming of antibiotic-resistant metabolome to antibiotic-sensitive metabolome. The synergistic effect is crucial in understanding metabolite-potentiated antibiotic-mediated killing. Importantly, CysJ is identified as a NOS-like protein in this bacterium.

## Supplementary Material

Supplemental Material

## Data Availability

The data that support the findings of this study are available from the corresponding author but restrictions apply to the availability of these data, which were used under license for the current study, and so are not publicly available. Data are however available from the authors upon reasonable request and with permission of participating local authority areas.
