# Supplementary material for: Synergy of alanine and gentamicin to reduce nitric oxide for elevating killing efficacy to antibiotic-resistant Vibrio alginolyticus
Source: Virulence. 2021 Jul 12;12(1):1737–53. doi: 10.1080/21505594.2021.1947447 (PMC8276662; doi:10.1080/21505594.2021.1947447)
Supplement: Supplemental Material [file KVIR_A_1947447_SM7616.doc]

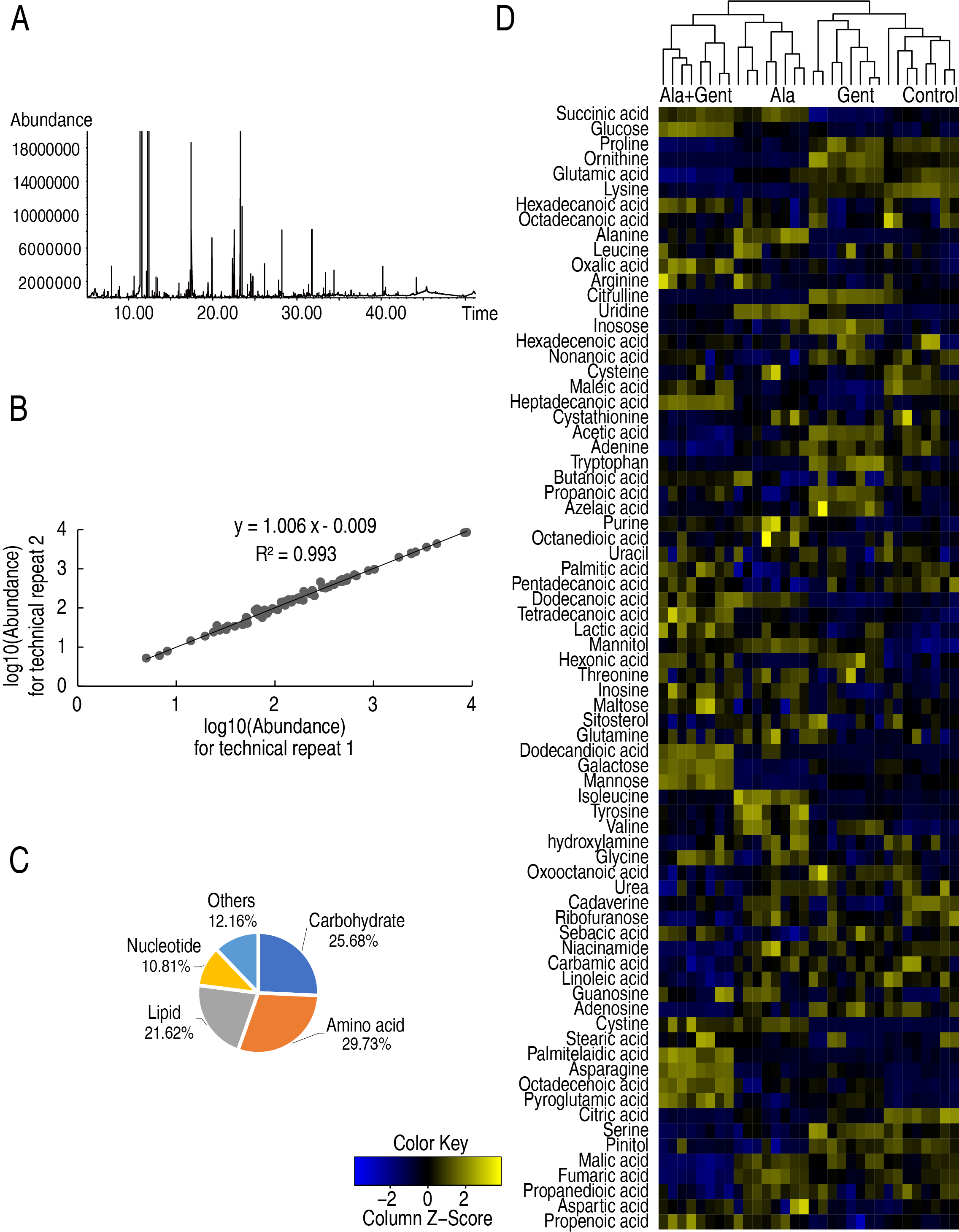


**Figure S1** Metabolomic profiling of *V. alginolyticus* ATCC33787. A. Representative total ion current chromatogram from control, Ala group, Gent group and Ala plus Gent group. B. Abundance of metabolites quantified in samples over two technical replicates is shown. Pearson correlation coefficient between technical replicates varies between 0.993 and 0.999. This plot shows the two replicates with the weakest correlation of 0.993. C. Category of all of the identified metabolites. D. Heat map of unsupervised hierarchical clustering of all metabolites (row). Yellow and blue indicate increase and decrease of the metabolites scaled to mean and standard deviation of row metabolite level, respectively (see color scale).


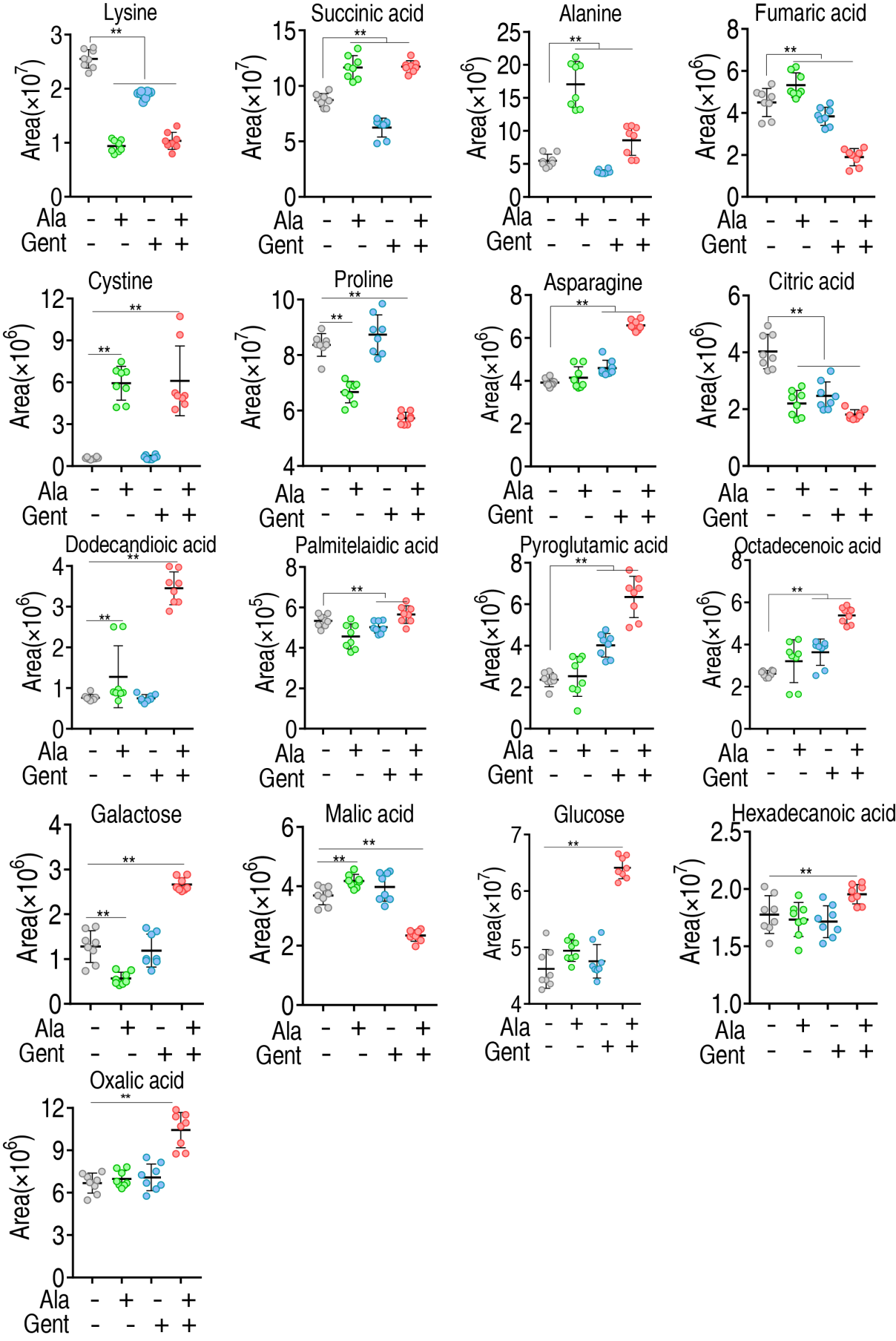


**Figure S2** Scatter plot of the differential metabolites in Ala group, the Gent group and the Ala plus Gent group. Results are displayed as mean ± SEM, and significant differences are identified **p < 0.01.


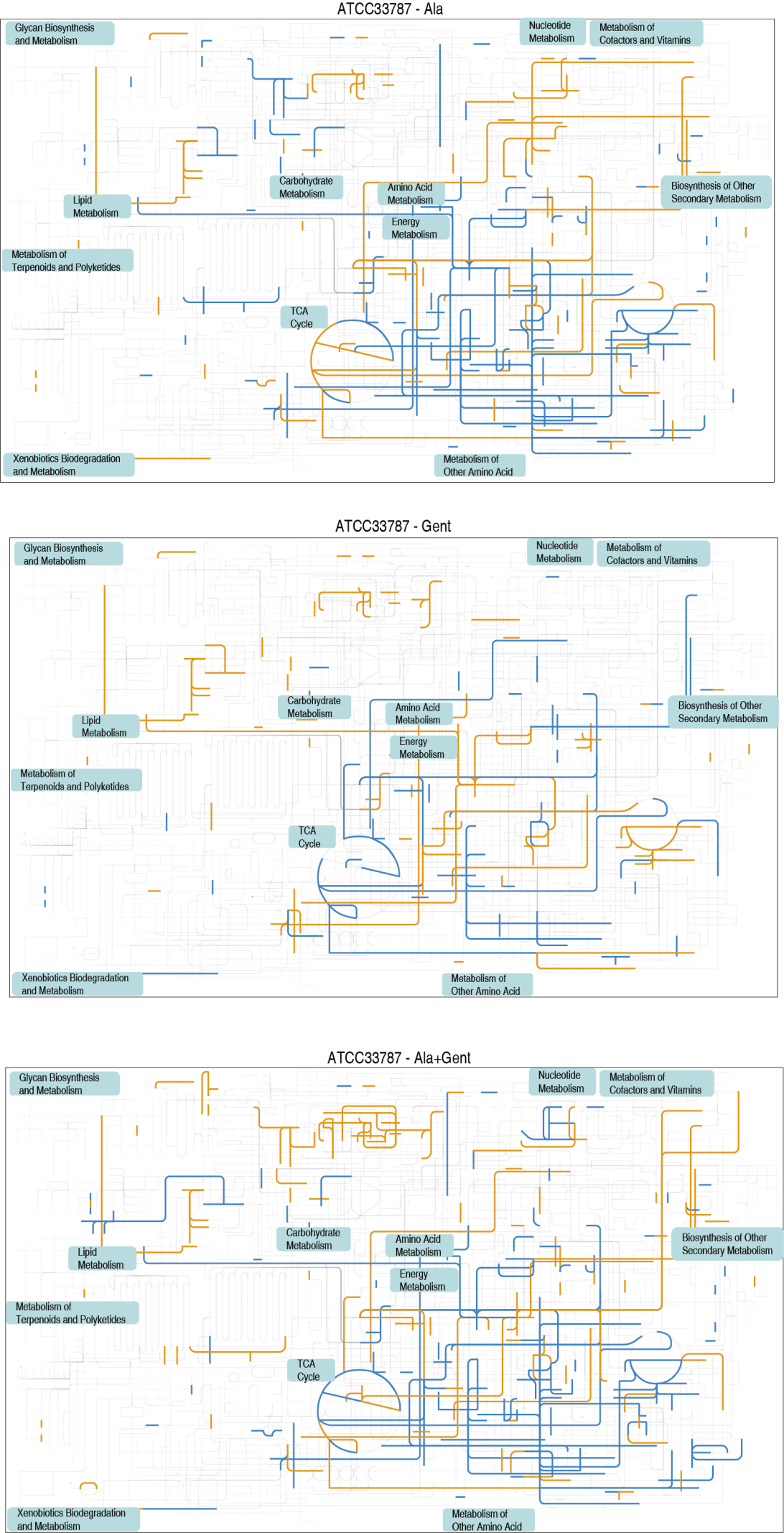


**Figure S3** iPath analysis. Blue and yellow lines represent decreased and increased metabolisms, respectively. 48 out of 51 significant metabolites (p<0.05) were submitted to online website (https://pathways.embl.de).


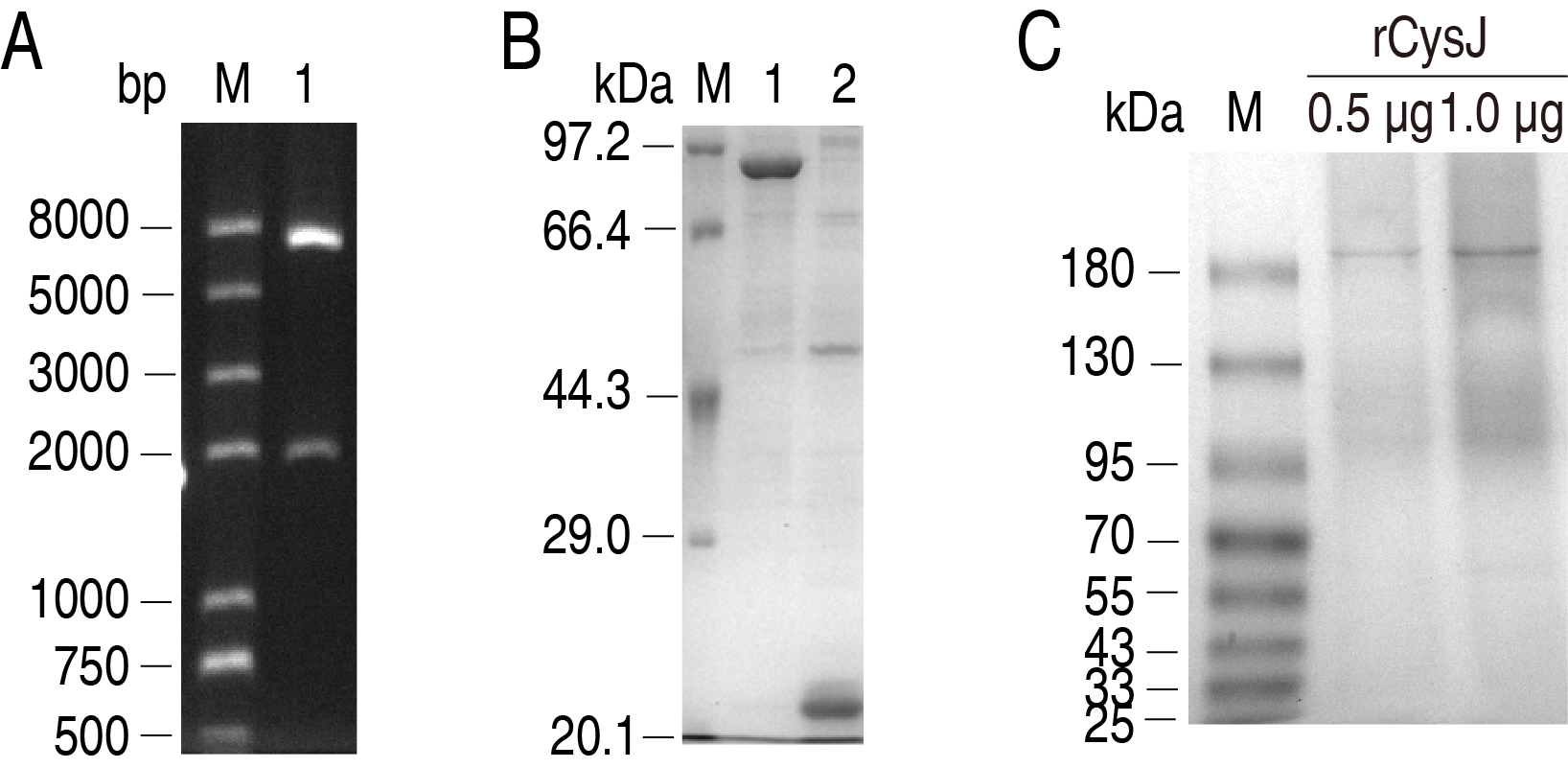


**Figure S4** Gene cloning and protein purification. A. Gene cloning of *cysJ* in ATCC33787. Lane M, DNA marker; Lane 1, double enzyme digestion of recombinant plasmid *rcysJ* (pET-32a vetctor). B. SDS-PAGE analysis for purify of purified rCysJ. Lane M, protein marker; Lane 1, rCysJ protein (pET-32a vetctor); Lane 2, pET-32a vetctor. C. Native-PAGE analysis for oligomeric state of rCysJ. Lane M, protein marker.


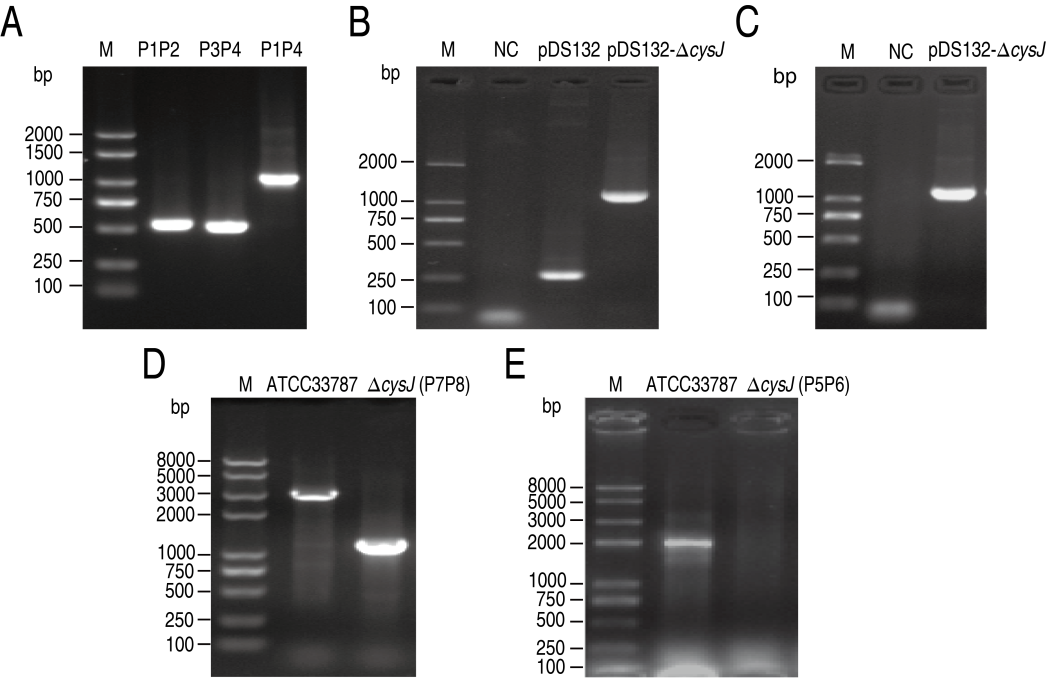


**Figure S5** Construction of *cysJ* mutant. A. Amplification of homologous recombinant fragment *cysJ*. Gel holes 1-3 represent the upstream (*cysJ*-P1/ *cysJ*-P2), downstream (*cysJ*-P3/ *cysJ*-P4), and fusion sequence (*cysJ*-P1/ *cysJ*-P4) of *cysJ* gene, respectively. B and C. Construction of pDS132-Δ*cysJ* recombinant vectors. The fusion fragment was successfully linked to pDS132 plasmid and transferred to *E. coli* MC1061 strains that were detected by PCR using pDS132F/R primers, -: negative control, +: pDS132 plasmid, Gel holes 1-3 represent pDS132-Δ*cysJ* recombinant vectors strains (B). The pDS132-Δ*cysJ* recombinant vectors were transferred to *E. coli* MFD-λpir strains, -: negative control, +: pDS132-Δ*cysJ* plasmid, Gel holes 1-3 represent pDS132-Δ*cysJ* recombinant vectors strains (C). D and E. Confirmation of Δ*cysJ*. Successful Δ*cysJ* mutant construction was detected by PCR using *cysJ*-P7/ *cysJ*-P8 (D) and *cysJ*-P5/ *cysJ*-P6 (E) primers for amplification. Gel holes 1-3 represent Δ*cysJ* mutant strains.

**Table S1** Primers used for *cysJ* gene cloning and qPCR of the *V. alginolyticus*

| Primers | Sequences (5’-3’) |
| --- | --- |
| *cysJ*- F1 | GCCATGGCTGATATCGGATCCATGTCTTTTCAAAAGAATGAGTATTCTCA |
| *cysJ*-R1 | GTGGTGGTGGTGGTGCTCGAGGTACACATCCCTCTGATAACGTTTCG |
| *cysJ*- F2 | ATTATGAAGCCCCCGCAAC |
| *cysJ*-R2 | CGTCAACAGTCTCAACCCCA |
| 16S rRNA-F | GGGAGTACGGTCGCAAGAT |
| 16S rRNA-R | GCTGGCAAACAAGGATAAGG |

* Homologous fragments were underlined.

**Table S2** Primers used for the construction and identification of the *V. alginolyticus* Δ*cysJ* mutant.

| Primer | Sequences (5’-3’) | Tm (°C) | Length (bp) |
| --- | --- | --- | --- |
| *cysJ*-P1 | AAAAAGGATCGATCCTGCCATGTTAGATATTCGATTTACTTAAA | 60 | 500 |
| *cysJ*-P2 | AAAAAGTCATTATATCACGACATCCCTATTCATTGC |
| *cysJ*-P3 | GATGTCGTGATATAATGACTTTTTCTACGGACAATAATAA | 60 | 500 |
| *cysJ*-P4 | ATCGCATGCGGTACCTGGGTTAGTGGTACACAACACGT |
| *cysJ*-P5 | ATGTCTTTTCAAAAGAATGAGTATT | 60 | 1872 |
| *cysJ*-P6 | TTAGTACACATCCCTCTGATAACG |
| *cysJ*-P7 | TGATTGATTTAGAGTCCGATAAATTCA | 60 | 3070 |
| *cysJ*-P8 | CCGTCTAGCCAAATCTCTGCATAAG |
| pDS132- F | CGGCAGGTATATGTGATGGGT | 60 | 278 |
| pDS133-R | CAACAAGCCAGGGATGTAACG |

* Homologous fragments were underlined.
